# Fabrication of innocuous gold nanoparticles using plant cells in culture

**DOI:** 10.1038/s41598-019-48475-9

**Published:** 2019-08-19

**Authors:** Sinilal Bhaskaran, Nilesh Sharma, Pooja Tiwari, Shree R. Singh, Shivendra V. Sahi

**Affiliations:** 1 Department of Botany, Fatima Mata National College, Kollam, Kerala 691001 India; 2 Department of Biology, Western Kentucky University, Bowling Green, KY 42101 USA; 3 Center for Nano Biotechnology and Life Sciences Research, Alabama State University, Montgomery, AL 36101 USA; 4 Department of Biological Sciences, University of the Sciences, Philadelphia, PA 19104 USA

**Keywords:** Nanoparticles, Molecular engineering in plants

## Abstract

Plant extracts and their different growth phases have been manipulated for the fabrication of nanomaterials, which can be an eco-friendly alternative to the chemical methods that produce hazardous by-products. However, practical difficulties in isolation of the nanoparticles obtained through biological methods and the scanty control that these methods allow over their shapes and sizes impose limitations in their utility. For the first time, we report here a versatile system using cell suspension culture of *Medicago sativa*, which ensures control over the reaction to regulate size of the particles as well as their easier recovery afterwards. Isolated nanoparticles were characterized for their shape, size and functions. The particles varied in shapes from isodiametric spheres to exotic tetrahedrons, pentagons and pentagonal prisms. They clearly demonstrated catalytic activity in the reduction reaction of methylene blue by stannous chloride. Interestingly, the cell culture-derived particles were found less cytotoxic to healthy human cell line HEp-2 while more cytotoxic to the cancer cell line 4T-1 in comparison to those synthesized through citrate method. However, when administered in mice, these nanoparticles elicited similar inflammatory responses as those produced by chemically synthesized counterparts. These results envisage the utility of these particles for various biological applications.

## Introduction

Conventional preparative techniques for synthesis of Gold Nanoparticles (AuNPs) employ physical and chemical methods. Physical methods including evaporation and laser ablation are expensive and laborious. On the contrary, chemical methods involve the reduction of a salt of gold (Au) with strong reducing agents, and are potentially hazardous to the environment either because of the toxicity of the reagents or by-products of the reaction^1,2^. The quest to find out an environmentally friendly and cost-effective method revealed the potential of inactivated biomass, plant extracts and intact plants to reduce gold from its ionic form (3+) to metallic (0) form and thereby favouring the process of particle formation^3–5^. Yet, the possibilities for manipulating biological techniques to produce viable products have not been studied well. One major obstacle faced is the standardization of the conditions for shape and size control in the background of scanty information about the biomolecules involved in the reaction. To some extent, separation of the particles from plant-borne contaminants also restricts further development of the technique. Understanding the mechanism of *in planta* nanoparticle fabrication with the associated transcriptomic/proteomic changes have also been variously reported^6–8^.

*Medicago sativa* is one of the various plant species well-studied for responses to heavy metal exposure^9,10^. A notable comparative study involving several species indicated that *M. sativa* possesses characteristics such as an increased uptake potential and reduced toxicity response toward heavy metals including gold, which may be advantageous for devising a plant-based fabrication system for nanomaterials^5^. However, growing plants under controlled conditions for fabrication and further processing for recovery of particles from the huge matrix of plant tissue is laborious. In this backdrop, utilization of cell suspension culture derived from the plant could be advantageous for both induction and isolation of the particles. Cell suspension culture allows the growth of intact cells under controlled conditions similar to bacterial broth culture. In order to test the feasibility of such a system, we initiated suspension cell culture of *M. sativa*, induced synthesis of gold nanoparticles using it and purified the particles. The product was further tested for their efficacy in chemical reduction and biological activity.

*M. sativa* has been shown to accumulate heavy metals^9^. Morphological and anatomical characteristics in addition to the molecular traits of a plant species may be implicated in the unusual potential for hyperaccumulation of heavy metals^11,12^. However, it is not known whether intact cells growing suspended in liquid medium can retain the above features. In order to verify this, we tested tolerance level of *Medicago* suspended cells to KAuCl_4_ before subjecting them to particle induction studies. AuNPs of different shapes and sizes are widely used in medicine and diagnostics, because of their unique physico-chemical, optical and surface properties^13,14^. Extensive studies are inevitable to assess their interaction with living systems before releasing them for therapeutic applications. Toxicity is one important aspect to be investigated, which is often dependant on the physical dimensions and surface chemistry of the AuNPs^15,16^. Cytotoxicity studies in human cells have shown that AuNPs are nontoxic up to 250 mM, while ionic gold shows obvious cytotoxicity at 25 mM^17,18^. Even though the AuNPs synthesized in cell culture are similar in their chemical and metallic nature to those synthesized by citrate method, their surface chemistry can vary since they are synthesized by the biomolecules inside *Medicago* cells. Hence the cellular fabrication of AuNPs in the present investigation was analysed by FT-IR to determine their surface chemistry and toxicity evaluation was carried out in Hep-2 and 4T-1 cell lines. As administration of commercially available AuNPs has been shown to trigger immunological responses (such as antibody and cytokine secretion) in mammalian hosts^16^, the cell-derived AuNPs were tested in this investigation for their effects using a murine model of inflammation.

## Results

### Tolerance of medicago cells to KAuCl_4_

As heavy metals are generally toxic to living cells, tolerance of *M. sativa* cells in suspension to KAuCl_4_ was determined by exposing the culture to its various concentrations (10–200 ppm). The highest concentration (200 ppm) affected cell viability by eliminating 25% cells from the culture after two weeks of continuous growth in the liquid medium (Supplementary Fig. 1A).

### Characteristics of the cell mediated nanoparticle synthesis

When the growing cell culture was supplemented with various concentrations of KAuCl_4_, the characteristic colour change took place in the reaction mixture from golden yellow to purple/violet after 18–20 h (Supplementary Fig. 1B,C). The reduction reaction was confined within the *Medicago* cells, since the residual liquid medium remained colourless after pelleting down the cells. MS medium when separately incubated with KAuCl_4_, in the absence of live cells, no reactions proceeded to form nanoparticles. The nanoparticles formed were identified as AuNPs by SEM-EDX of the cell lysate (Supplementary Fig. 2A,B) and their intracellular fabrication was ascertained by TEM analysis of ultrathin sections (Supplementary Fig. 1D).

### Effect of KAuCl_4_ concentration on particle size

**Figure 1:**
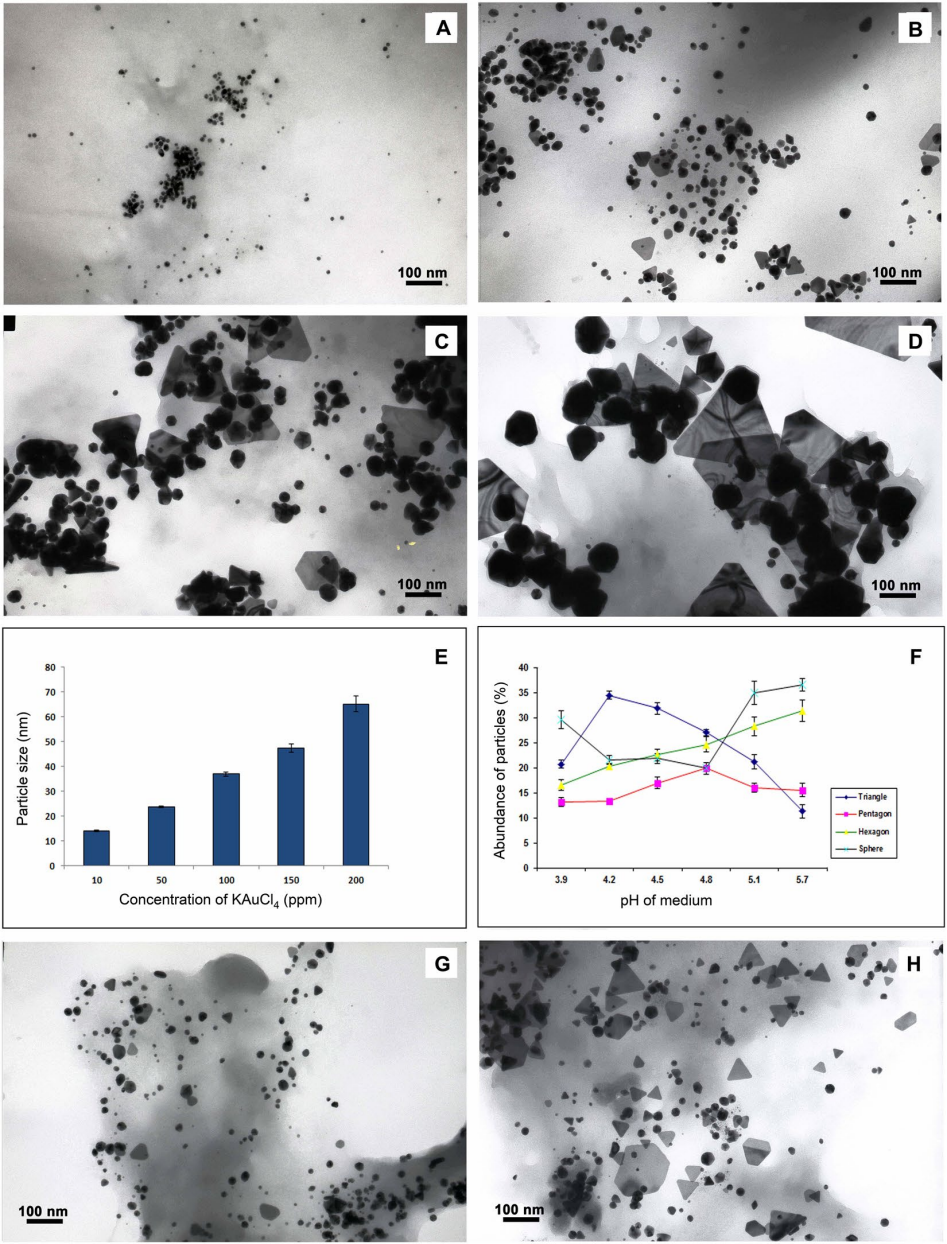
Influence of reaction conditions on the geometry of AuNPs synthesized by the cells. Images (**A**–**D**) show variation in the size of nanoparticles synthesized under10, 50, 100 and 200 ppm of KAuCl_4,_ respectively. (**E**) Displays the size of particles plotted against different concentrations of KAuCl_4_ used for induction. Values are means of 1,000 particles ± SE. Since p value is lower than 0.05 and the value of F is above F Crit in ANOVA, the difference between the groups is significant. (**F**) Indicates the abundance of AuNPs of different geometrical shapes plotted against changing pH of the medium. Images (**G**) and (**H**) display particles synthesized in the culture medium at pH 5.8 and 3.9, respectively. In ANOVA, p value is lower than 0.05 and F value is less than F Crit validating insignificant difference between the groups. Values are means of 1,000 particles ± SE.

Spheres, triangles, pentagons and hexagons were the major types of particles found distributed throughout the samples analysed (Fig. 1A–D). A small proportion of rods and rhomboids were also found (Supplementary Fig. 3). Approximately, 95% of nanoparticles induced under the treatment of 10 ppm KAuCl_4_ were spherical whereas other particle shapes (triangles, pentagons and hexagons) increased in proportion to an increase in KAuCl_4_ concentration. A small proportion of spherical particles (15–20 nm) were common to all treatments. A linear progression in particle size was evident in accordance with the increase in KAuCl_4_ concentration (Fig. 1A–E). Diameter of particles formed under different concentrations of KAuCl_4_ ranged between 15–75 nm. In addition to the solid particles, translucent triangular and hexagonal nanoplates were also observed in samples treated with higher concentrations of KAuCl_4_ (Fig. 1C,D, Supplementary Fig. 3). Even though the effect of pH was studied by altering pH of the nutrient medium (at KAuCl_4_ 100 ppm), its effect was not found significant (Fig. 1F–H).

### Isolation of AuNPs from medicago cells

**Figure 2:**
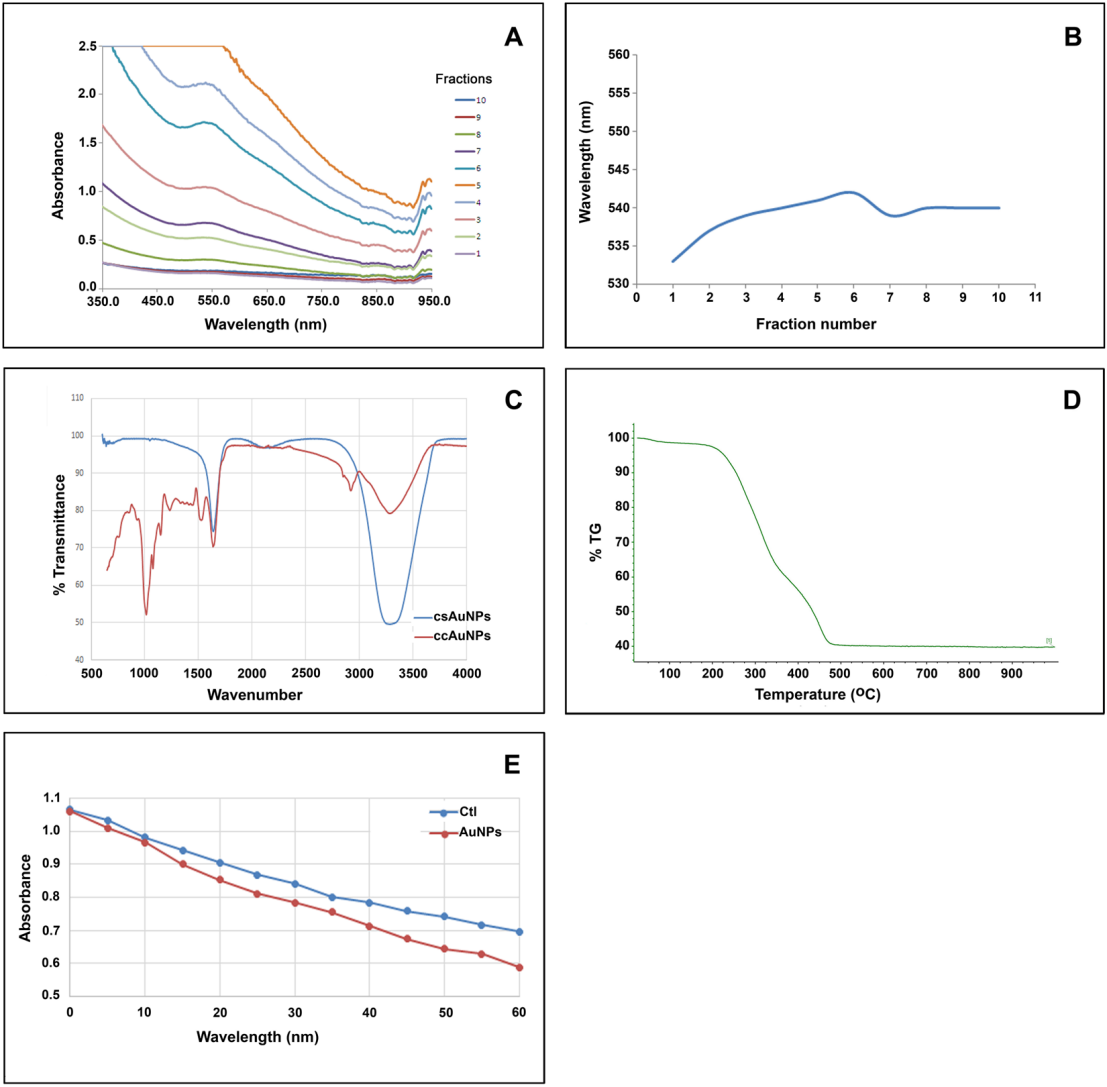
Purification and characterization of AuNPs from the cells of *M. sativa*. (**A**) UV-Vis absorbance spectrum of the particles eluted from the gel permeation column showing peak around 540 nm. (**B**) Distribution of absorbance peaks of the fractions eluted from gel permeation column indicating size similarity among the particles. (**C**) FT-IR transmittance peaks of ccAuNPs (compared with csAuNPs) show the presence of aliphatic alcohol (1480–1405, 1075–100 cm^−1^), alkyl (2990–2855, 1485–1415 cm^−1^), hydroxyl/amino groups (3540–3200, 1205–885 cm^-1^) on particle surface. (**D**) TGA analysis plot showing the disintegration of these functional groups at and onward 500 °C. Catalytic activity of the isolated particles exhibited in the reduction of methylene blue by SnCl_2_. (**E**) Absorbance at 660 nm of the reaction with ccAuNP as catalyst, plotted against the respective control; reaction over a period of 1 hr shows the enhancement rate of 33%.

AuNPs synthesized by the cells were separated from plant borne contaminants, medium components and unreacted KAuCl_4_ by passing through Sephadex G100 column. Particles migrated through the column together as a purple band, facilitating the monitoring of sample migration and fraction collection. Eluted fractions produced absorbance peak around 540 nm which is characteristic to AuNPs due to the surface plasmon band. Height of the peaks varied according to colour intensity of the fractions (Fig. 2A). Minimal shift in absorption maxima was observed among various fractions eluted, which was supposed to be due to similarity in size of the particles (Fig. 2B). Almost all fractions showed a characteristic absorption peak at NIR region, indicating the abundance of triangular or polyhedral particles rather than spherical. This was later confirmed by fraction-wise analysis in TEM. FT-IR scans of these fractions showed the presence of aliphatic alcohol, alkyl, hydroxyl/amino groups on the surface (Fig. 2C). Thermo Gravimetric Analysis of air dried nanoparticles demonstrated significant weight loss between 225 °C and 475 °C, indicating the disintegration of these functional groups at this temperature range (Fig. 2D).

### Morphological characterization of AuNPs using stereo TEM

**Figure 3:**
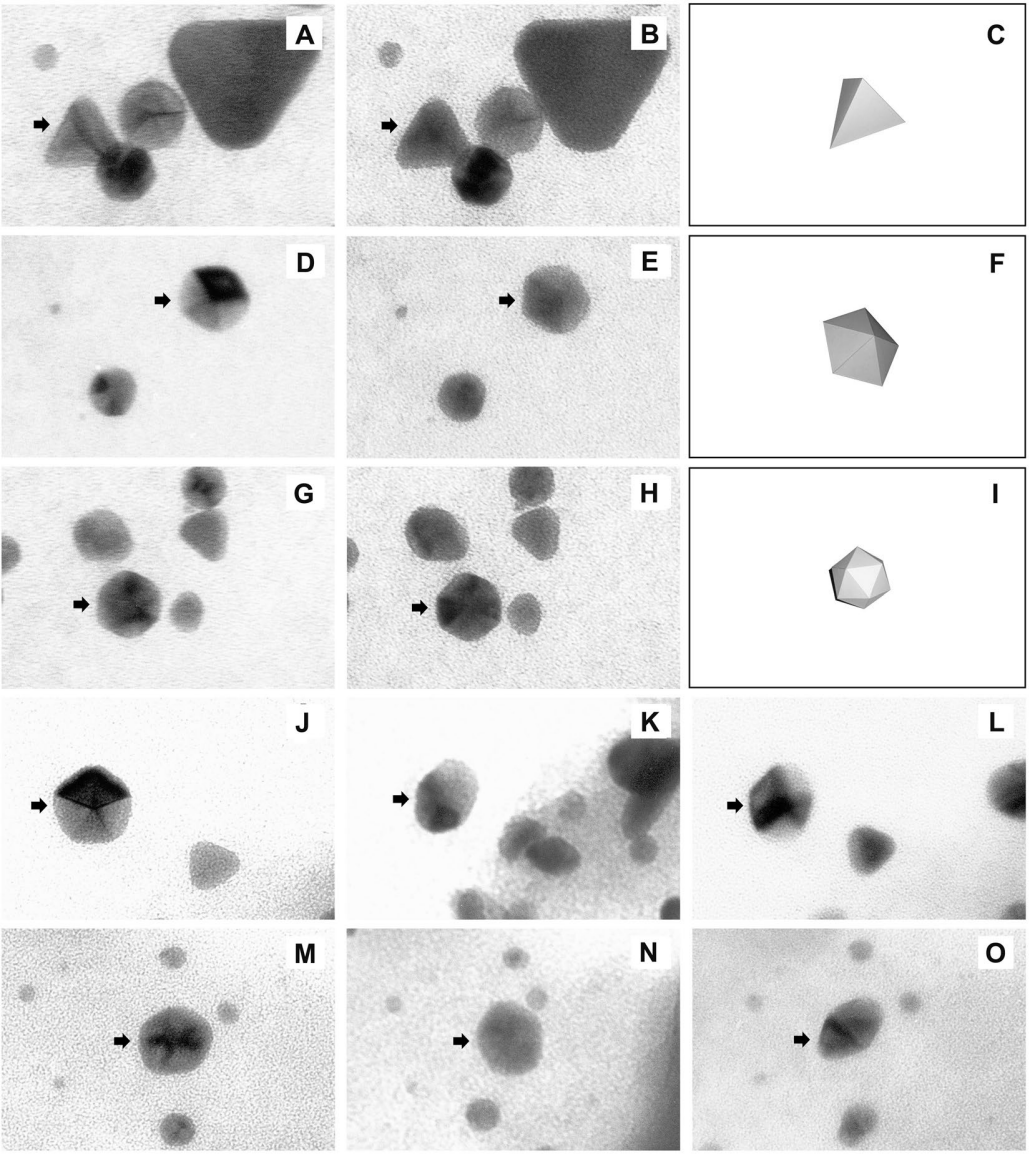
Analyses of three-dimensional features of isolated AuNPs by stereo TEM reveal true shapes of the particles. Images of triangle (**A**), pentagon (**D**) and hexagon (**G**) captured at +5° have different shading patterns in their respective images captured at −5° (**B**,**E**,**H**) identifying them as tetrahedron (**C**), pentagon (**F**) and pentagonal prism (**I**). Images of two-sided pentagonal pyramids at +45° (**J**,**M**), 0° (**K**,**N**) and −45° (**L**,**O**) illustrate how the particles can be identified as rhomboids in regular TEM. Arrows indicate the particles under observation.

Stereo images captured in a regular TEM displayed differential shading patterns according to the angle of rotation, which was due to the differential scattering of the electron beam on their faces. This analysis helped to distinguish between triangular plates and tetrahedrons which appeared merely as triangles in regular TE micrographs (Fig. 3A–C). Pentagons were discovered to be pentagonal prisms (Fig. 3D–F) whereas those appeared as hexagons were identified as icosahedrons under our image capture technique based on stereomicroscopy (Fig. 3A–I). Pictures taken at three consecutive angles of 45° intervals proved that the particles appearing as rhomboids in regular TEM images were not true rhomboids, but pentagonal pyramids having two five sided opposite faces (Fig. 3J–O).

### Assessment of catalytic activity of the isolated nanoparticles

Isolated nanoparticles demonstrated catalytic activity in the reduction reaction of methylene blue by stannous chloride. Progression of the reaction was monitored by observing the height of absorbance peak at 660 nm. Readings taken for 1 h at 5 min intervals indicated successive reduction in absorbance in test as well as control groups (Fig. 2E). Addition of nanoparticle suspension to the reaction mixture causing about 32% increase in the reaction rate is a clear proof of the catalytic activity (Fig. 2E).

### Interactions of the AuNPs with animal cells *In Vitro* and *In Vivo*

**Figure 4:**
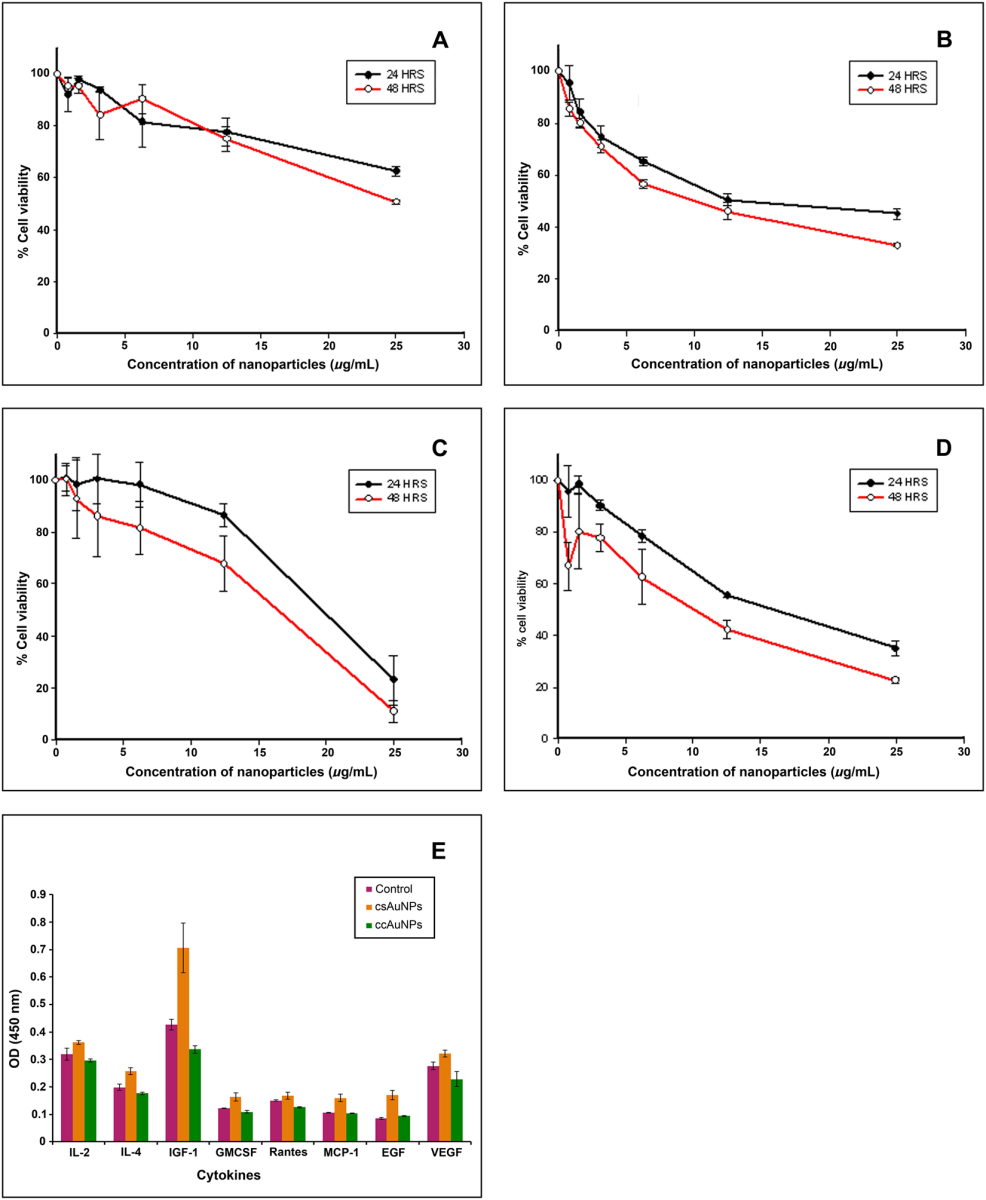
AuNPs synthesized by the cell culture (ccAuNPs) has cytotoxicity and immune reactivity comparable to those synthesized by chemical synthesis, citrate reduction (csAuNPs). (**A**) Cell death induced due to the toxicity of ccAuNPs on HEp-2 cells. (**B**) Cell death induced due to the toxicity of csAuNPs, plotted as percentage of cell viability against the concentrations of nanoparticles used. (**C**) Cell death in 4T-1 cells exposed to ccAuNPs. (**D**) Cell death in 4T-1 cells exposed to csAuNPs. (**E**) Serum levels of cytokines: IL-2, IL-4, IGF-1, GMCSF, Rantes, MCP-1, EGF and VEGF in mice following exposure of csAuNPs (orange bar), ccAuNPs (green bar) and control (no nanoparticles, purple bar). The variations between groups are statistically not significant (t-test; p > 0.05).

Toxicity of the isolated nanoparticles when tested by MTT assay, the cell culture-synthesized nanoparticles (ccAuNPs) inhibited approximately 50% human epithelial cells (HEp-2) while chemically synthesized nanoparticles (csAuNPs) induced inhibition in 60% of cells in the same cell line (Fig. 4A,B). Interestingly, effects on cancer cell line 4T-1 were significantly different for both types of nanomaterials: ccAuNPs caused 70–90% cell death while csAuNPs had mortality rate of 50–70% for 4T1 cancer cell line at the inhibitory concentration, 25 µg/ml, after 24 and 48 h of incubation (Fig. 4C,D). ccAuNPs were tested further for their inflammatory responses *in vivo*. The levels of cytokines and growth factors, including IL-2, IL-4, IGF-1, GMCSF, Rantes, MCP-1, EGF and VEGF (Fig. 4E) in mice were not significantly different (p > 0.05) between two groups of mice challenged with 200 µL of ccAuNPs and csAuNPs each, and were comparable to the unchallenged control. Expression pattern of few genes associated with inflammatory responses further testifies the efficacy of ccAuNPs (Supplementary Fig. 4).

## Discussion

The cells of *M. sativa* demonstrate tolerance to higher concentrations of KAuCl_4_, as evidenced by their low percentage of death. Even though inhibition of root growth by KAuCl_4_ was reported earlier in plants grown in a hydroponic set up^5^, those concentrations had no significant effect on the cell viability in suspension culture described herein. The AuNPs synthesized were found accumulated inside the cells on the analysis of thin sections by TEM, validating the intracellular fabrication mediated by the internal milieu of the cell. This, in turn, rules out the possibilities for involvement of any of the components of the nutrient medium. Likewise, internal accumulation of AuNPs was noticed in *Sesbania drummondii* when whole plants were treated with KAuCl_4_^4^_._

Analysis of nanoparticle morphologies revealed a significant positive correlation between the feeding concentration of KAuCl_4_ and the size of the AuNPs formed. Statistical analysis (ANOVA) clearly indicates the significant variations (p < 0.01) among different groups. Particles generated under different concentrations of KAuCl_4_ correspond to the size range of particles used for various diagnostic and therapeutic purposes in medicine^13–15^. Another interesting observation was the formation of triangular and hexagonal nanoplates along with AuNPs in higher concentrations of KAuCl_4_. In chemical synthesis when the reduction reaction is carried out in presence of CTAB, inhibition of some facets of seeds occurs leading to two-dimensional deposition of Au atoms, favouring the formation of nanoplates. On the contrary, secondary homogenous nucleation which directs formation of multifaceted particles decreases^19,20^. In case of plant mediated AuNP synthesis, higher concentrations of KAuCl_4_ were also observed to be favouring plate formation. Such a correlation between the morphology and KAuCl_4_ has not been observed in CTAB-mediated synthesis, and hence the plant-mediated process of nanoparticle synthesis can be speculated as following a different process. Such plates are useful in optical bio-sensing and surface-enhanced Raman spectroscopy because of the high local electric field gradients generated by their sharp edges under illumination^21^. The effect of pH is well established in chemical synthesis of AuNPs. Accordingly, low pH is preferred because of its utility in governing the shape and preventing aggregation^21^. Even though change in pH brought about change in shape of the particles to some extent, the effect was not found statistically significant (p > 0.05). Since nanoparticle formation is occurring inside the cells, pH of the cellular environment may not correspond to the changes in the medium after an equilibrium. Earlier studies revealed that acidic pH (nearly 3) was conducive to the synthesis of polyhedral particles in case of *M. sativa* extract^10^; pH 3.9 had induced formation of triangles in case of the whole plant^5^.

A major hurdle in plant-mediated AuNP synthesis is the recovery of the particles from the reaction mixture after synthesis. We tested size exclusion chromatography for separating the plant cell-borne materials, remnants of the medium and unreacted KAuCl_4_ from the AuNPs^22,23^. Fractions eluted from the column showed differences in height of the peak at 540 nm which indicated differences in particle distribution^24,25^. It was confirmed later by fraction-wise TEM analysis. Catalytic activity is one of the attributes in AuNPs catering their wide range of industrial applications. Demonstration of the activity in AuNPs isolated from the cells by the reduction reaction of methylene blue testifies their usage as catalysts^26^. The surface bound chemical groups predicted by FT-IR and TGA analysis indicated the involvement of cellular biomolecules in the particle synthesis. Both primary and secondary metabolites are known to be associated with the process of nanoparticle synthesis^27^. Though a plethora of plant-based studies on AuNPs synthesis were reported in recent times, the involvement of plant-borne reducing agents in the mechanism is not yet identified.

Nanoparticles of different morphologies were identified with the help of TEM. However, lack of three-dimensional details in the conventional transmission electron micrographs imposes difficulties in the precise characterization of particle shape. Since optical properties of the particles are dependent on their three-dimensional features, solving the 3D structure is critical for applications involving light scattering^28^. Techniques such as high resolution TEM^1^ Multispectral Chiral Lens (MCHL)^29^, SAED^30^ and XRD^31^ have been used for this purpose. Stereomicroscopy is a simple technique, much useful in this context^32,33^. It utilizes pairs of images taken at orientations differing by an angle greater than 5°. On comparative analysis between the pictures captured at consecutive angles in regular TEM, the true 3D shapes of the particles were revealed. Thus, stereomicroscopy was proved to be an effective tool for resolving the 3D structures of polyhedral nanomaterials.

Toxicity testing is a prerequisite for determining the suitability of the AuNPs for various diagnostic and therapeutic applications. Chemically synthesized gold nanoparticles (csAuNPs) have been generally accepted as non-toxic materials to animal cells based on the results of appropriate methods of testing^17,34^. It is judicious to apply similar standards in the assessment of toxicity of cell culture-synthesized nanoparticles (ccAuNPs). Interestingly, the cell culture-derived particles were found to be less cytotoxic to healthy human cell line HEp-2 while more cytotoxic and to the cancer cell line 4T-1 in comparison to those synthesized through citrate method (p < 0.5). Cell culture-derived AuNPs were further verified for their inflammatory responses *in vivo*. Tracking peripheral inflammatory markers such as cytokines is a common practice to evaluate the inflammatory response caused by a foreign substance including nanoparticles^35^. Proinflammatory and anti-inflammatory cytokines are mediators of an inflammatory process that precedes a host of chronic conditions in humans. Since the levels of cytokines and growth factors in mice were either comparable to the control or to csAuNPs (p > 0.05 in t-test) group. Results of transcript analysis by real-time PCR also supported this pattern (Supplementary Fig. 4). As immune response largely depends on the size, shape and surface charge of nanoparticles, a systematic examination is required to reveal the influence of these parameters on inflammatory or specific immunogenic responses and thus we included *in vivo* response test in our investigation^36,37^.

The cell culture method described herein for AuNP synthesis is advantageous over the methods based on cell lysates mainly because of the greater control over particle shape and size, and the easy of recovery of the particles from the reaction mixture. However, time needed for completion of the reaction is almost similar in both cases. Even though cell culture experiments displayed a tendency to modulate particle shape based on the changes in pH of the medium, this effect was not found statistically significant.

## Methods

### Continuously growing plant cell culture

Hypocotyl regions dissected out from one-week-old seedlings of *M. sativa* (germinated on water agar medium) was inoculated on MS medium^38^, pH 5.7, supplemented with 30% sucrose, 0.8% agar, 0.2 mg/L IAA and 0.2 mg/L BA. After two weeks, the callus mass derived from the explant was transferred to liquid medium with the same composition. Conical flasks containing 1/5^th^ volume of the culture was grown on an orbital shaker with 100 rpm at 27 °C. Callus clumps remaining in the medium were removed after two days. After one week, cell suspension was collected, discarding the clumps of callus remaining in the bottom. The process of clump removal from the growing suspension was repeated three times to achieve a homogenous stock of cell suspension. The single cell nature of the culture was confirmed by microscopic examination. Sub culturing was done once in every two weeks by spinning down cells at 2000 rpm for 10 min and adding fresh medium following the removal of the old one.

### Testing KAuCl_4_ tolerance of Medicago cells in culture

To determine the IC_50_ of KAuCl_4_, the filter sterilized stock solution of KAuCl_4_ (prepared in deionized water) was added at different concentrations (10–200 ppm) to 20 mL culture having a cell density of 20,000 cells/mL and incubated on the shaker for 2 weeks. Culture aliquots (1 mL) were collected at an interval of 48 h, spun at 2000 rpm for 10 min, stained with phosphate buffered Trypan Blue and dead cells were counted using a haemocytometer. Percentage of cell death was determined from the total cell number.

### Synthesis of AuNPs

KAuCl_4_ was supplemented to the cell culture at the rate of 10–200 ppm and incubated on a shaker for 24 h in darkness. Aliquots (1 mL) of the culture were collected in Eppendorf tubes and centrifuged for 2 min (14,000 rpm) and the medium was removed. Same amount of nanopure water was added to wash away traces of medium and the unreacted gold. After 5 repeated washes, the cells were allowed to re-suspend in 100 μL of water and lysed using a sonicator for 2 min constituting an amplification cycle of 80% intensity with a pulse for 20 sec and off time for 10 sec.

### Recovery of AuNPs from plant cells

Cells harbouring the nanoparticles were collected by centrifugation and lysed by sonication as described above. Cell debris was separated by centrifugation at 200 g (1400 rpm) for 2 min. Supernatant (1 mL) was then loaded on to Sephadex G100 (medium) column with a bed volume of 12 cm × 1.8 cm (height × diameter) equilibrated with water. Fractions of 1 mL were eluted with water from the column at a flow rate of 1 mL/min. Collected fractions were scanned on UV-Vis spectrophotometer (Perkin Elmer, Lambda XLS+) at 200–900 nm. SDS was added to a final concentration of 0.1%, vortexed briefly and centrifuged at 6,000 g (7,900 rpm) for 30 min at 4 °C and the supernatant removed followed by the addition of fresh nanopure water. Efficiency of gel filtration and the washing step in removing plant cell borne contaminants was verified by testing the total protein content by Bradford test and the presence of nucleic acids by running on 1% agarose gel. Individual fractions were scanned on FT-IR spectrometer (Perkin Elmer, Spectrum 100) at 600–4000 nm to detect the presence of particles.

### Assay for catalytic activity

Catalytic activity of the particles isolated was tested in the reduction reaction of methylene blue by SnCl_2_ in presence of SDS. 100 μL of nanoparticle suspension in water (OD 1 at 550 nm) was added to 3 ml SDS (0.01 M), 10 μL methylene blue (0.5 mM) and 50 μL SnCl_2_ (0.05 M). Absorbance at 500–800 nm at 5 min intervals was recorded to monitor the reaction rate. A control reaction was run in parallel adding 100 μL water instead of the nanoparticle suspension.

### Cytotoxicity testing of AuNPs

Toxicity of the isolated nanoparticles on human epithelial type 2 cell (HEp-2: ATCC CCL-23) and mouse mammary gland tumour cell line (4T-1: ATCC CRL-2539) was tested by MTT assay. HEp-2 cells were maintained in Minimum Eagle’s Medium (MEM) supplemented with 10% foetal bovine serum (FBS), L-glutamine (2 mM), PKS [penicillin (75 U/mL), kanamycin (100 µg/mL) and streptomycin (75 µg/mL)]. 4T-1 cells were maintained in Dulbecco’s MEM Glutamax supplemented with 10% FBS and 1% Antibiotic-Antimycotic (100x). Cells were grown to achieve a density of 17,000 cells per well in 96 well tissue culture plates. Following overnight incubation, the culture medium was replaced by fresh medium with serial dilutions of nanoparticle starting from 0.7 to 25 μg/mL. Percentages of cell viability after 24 and 48 h incubation were determined by the assay.

### Electron microscopy

Sonicated samples (5 μL) were loaded on 100 mesh formvar coated copper grid. Particles were allowed to settle for 5 min and the excess cell lysate was blotted and dried on a hot plate at 50 °C for 10 min. Samples were viewed at 100 kV and images captured at 66, 000 X by 120 CX TEM (JEOL JEM). Scanned images (1200 dpi) of the particles were subjected to morphological analysis using the software Iridium Ultra version 1.4 (www.ixrfsystems.com). For unravelling the three dimensional features of the particles, pictures were taken at 100,000X, rotating the grid at different angles. Energy Dispersive X-ray Spectroscopy (EDS) of the cell lysate was done by loading the sample on carbon ribbon and viewed at an accelerating potential of 20 kV under high vacuum mode with backscatter detector in JSM-5400LV scanning electron microscope (JEOL) equipped with IXRF EDS system with Moxtek AP3.3 light element entrance window.

### Cytokine detection by ELISA

Lab mice C/57-BL6/J (10-week-old, female mice, 5 per group) were intra-peritoneally injected with 200 µL (333 μg/kg body weight) of cell culture-synthesized nanoparticles (ccAuNPs) and chemically synthesized particles (csAuNPs) each. Control was maintained with 200 µL of PBS. Blood was harvested after 15 days of exposure in mice. Blood was stored overnight before processing for the serum. Serum samples were further processed using Custom Mouse ELISA Strip (Signosis, Santa Clara, CA), and absorbance was measured by Synergy plate reader at 450 nm. The testing protocol “Assessment of Plant-derived, Ecofriendly Gold Nanoparticles using an Animal System” was approved by Western Kentucky University’s Institutional Animal Care and Use Committee (Animal Welfare Assurance # A3558-01) and was assigned the following designation (16-09). The approved protocol remains valid from 12/08/2016 to 12/07/2019.

### Statistical analysis

Statistical analyses were carried out in Microsoft Excel 2010. Significance of the results of particle size and shape analyses was verified by ANOVA. Results of the tolerance test of plant cells to KAuCl_4_, toxicity testing of AuNPs in animal cell lines, cytokine detection and real-time PCR in mice were analysed for significance using t-test. Level of significance was kept at 0.05 for all the analyses.

## Supplementary information

Supplementary Figures
