# Supplementary material for: Fabrication of innocuous gold nanoparticles using plant cells in culture
Source: Sci Rep. 2019 Aug 19;9:12040. doi: 10.1038/s41598-019-48475-9 (PMC6700120; doi:10.1038/s41598-019-48475-9)
Supplement: Supplementary file 1 — Supplementary Figures [file 41598_2019_48475_MOESM1_ESM.pdf]

Fabrication of innocuous gold nanoparticles using plant cells in culture

Bhaskaran Sinilal[1], Nilesh Sharma[2], Pooja Tiwari[3], Shree R. Singh[3] and Shivendra V. Sahi[4,*]
[1]Department of Botany, Fatima Mata National College, Kollam, Kerala, India- 691001
[2]Department of Biology, Western Kentucky University, Bowling Green, KY 42101, USA.
[3]Center for Nano Biotechnology and Life Sciences Research, Alabama State University, Montgomery, AL 36101, USA.
[4]Department of Biological Sciences, University of the Sciences, Philadelphia, PA 19104, USA; *E – mail: s.sahi@usciences.edu

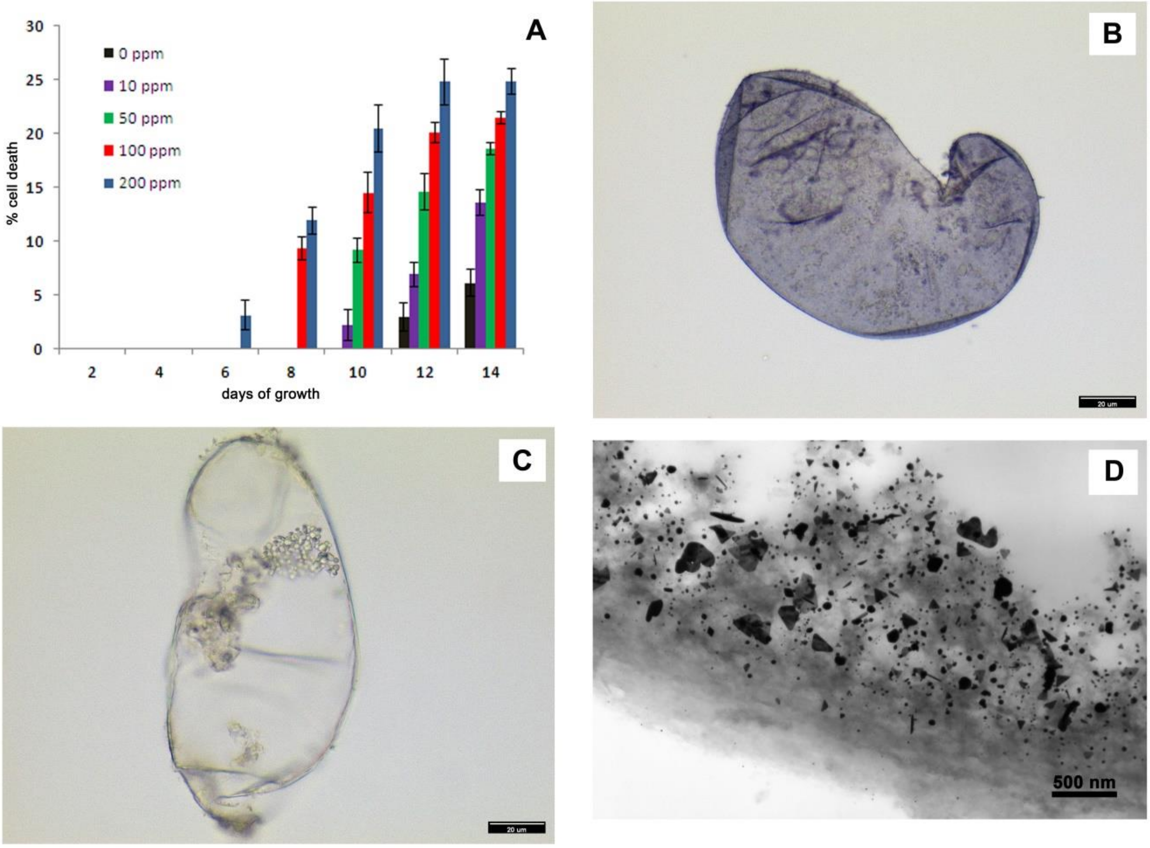

Figure 1. Interaction of $KAuCl_4$ with the cells of *M. sativa*. Percentage of death in the cells of *M. sativa* treated with four different concentrations (10, 50, 100 and 200 ppm) of $KAuCl_4$ plotted against that of the untreated (0 ppm) control group. (A). Color change in cells treated with $KAuCl_4$ (B) and the untreated control (C) showing its absence (observable in all the concentrations of $KAuCl_4$ tested; however the color intensities increased from 10 ppm to 200 ppm which is assumed to be due to the increase in number and size of the particles distributed in the respective samples). Ultra-thin section of cell treated with $KAuCl_4$ showing AuNP accumulation inside the cytoplasm (D). Values of cell count are means of three biological replicates and two technical replicates each and plotted with ±SE. Significance analysis by t-test showed a value higher than 0.05.

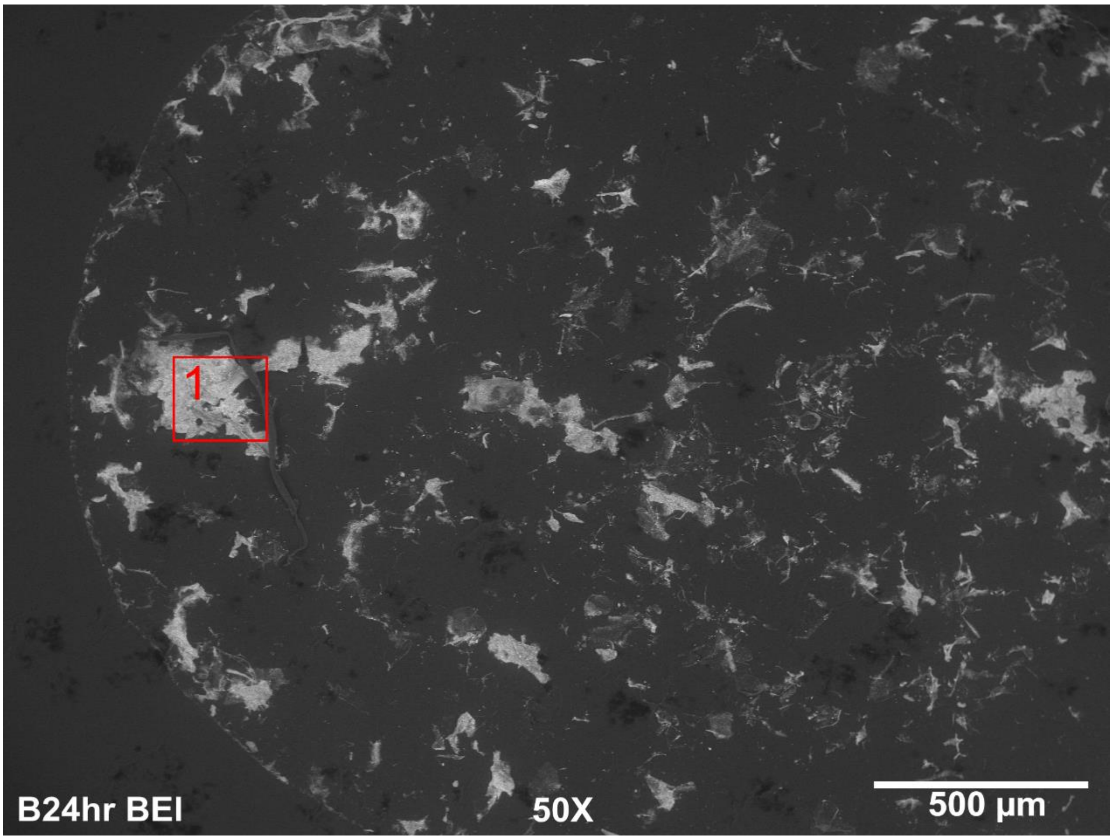

Figure 2A. Image from SEM showing AuNPs distributed in the cell lysate. Numbered box indicates the area scanned for detecting the relative concentration of different elements.

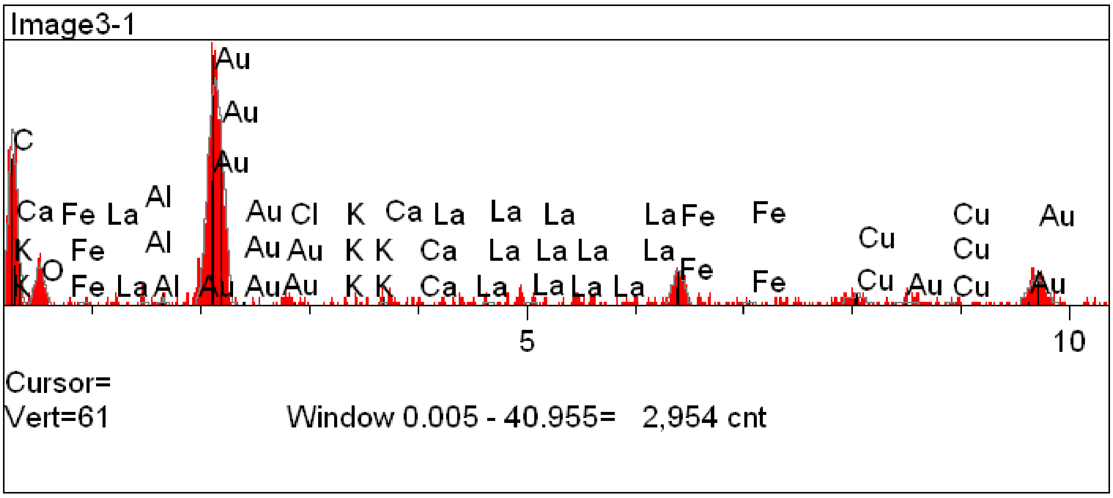

Figure 2B. EDX spectrum showing the highest peak of gold confirming the nature of AuNPs synthesized by the suspension cells of *M. sativa*. Carbon peak development is due to the carbon tape used for loading the sample.

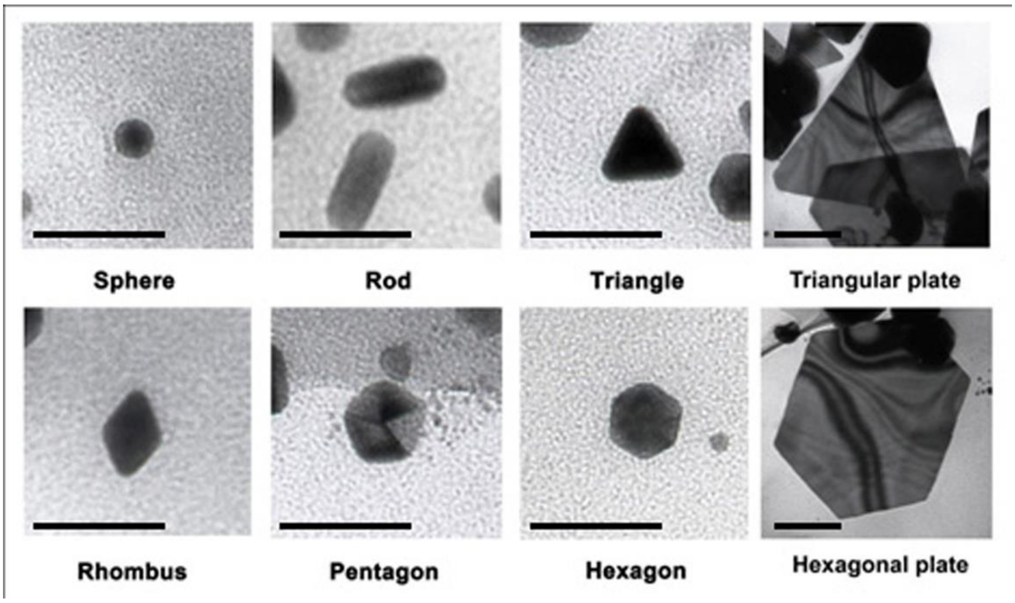

Figure 3. AuNPs of different geometries synthesized in the suspension cells of *M. sativa*. Scale bars are of 100 nm.

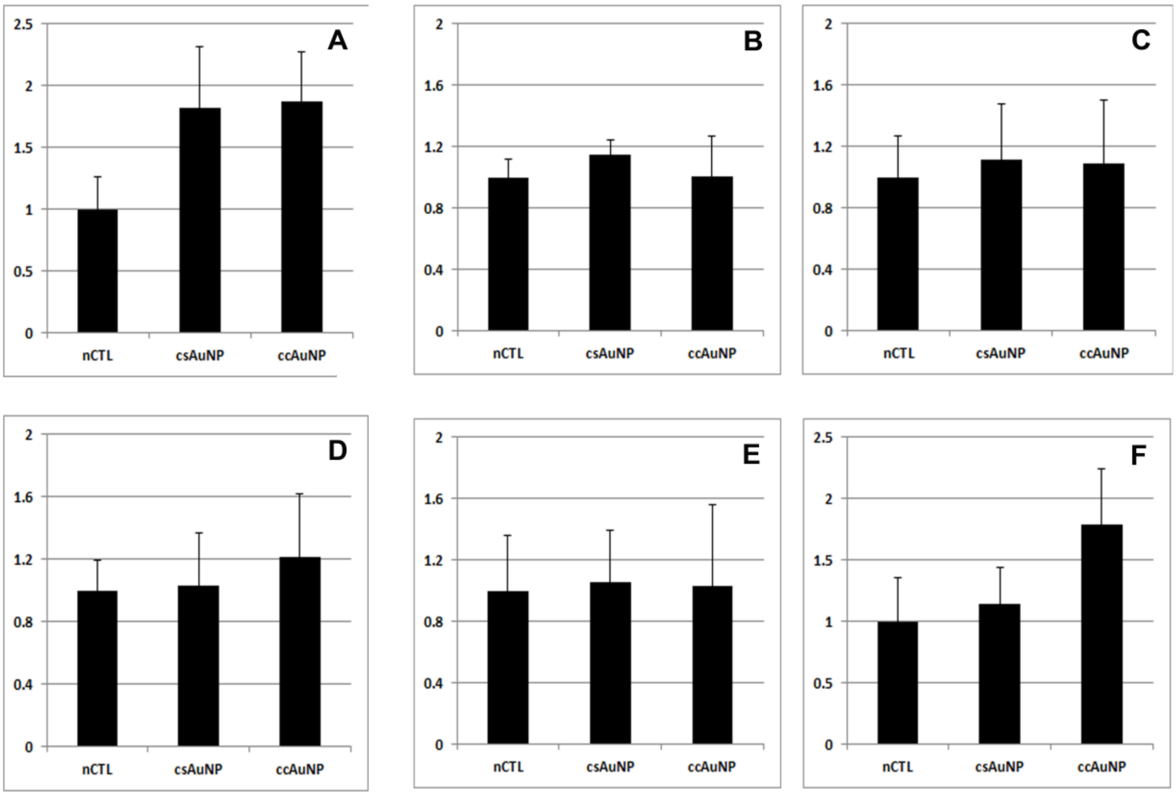

Figure 4. Expression levels of genes (analyzed by qPCR) associated with immune response in mice subjected to ccAuNPs show comparable effects to those of csAuNPs. Relative expression levels of *MCP1* (A), *TGFβ* (B), *TNFα* (C), *IFNγ* (D), *IL1α* (E) and *IL10* (F) of the AuNP treated groups are plotted against those of the normal control (nCTL). No significant differences (t-test; p>0.05) between the two treatment groups were recorded.
